# Collective case formulation in situations of violent radicalization: A critical perspective in training

**DOI:** 10.1177/13634615221134932

**Published:** 2023-01-11

**Authors:** Janique Johnson-Lafleur, Yann Zoldan, Rochelle L. Frounfelker, Cécile Rousseau

**Affiliations:** 1 Department of Anthropology, 5620 McGill University, Montreal, Quebec, Canada; 2 Department of Health Sciences, 14661 Université du Québec à Chicoutimi (UQAC), Chicoutimi, Quebec, Canada; 3 Department of Community and Population Health, College of Health, Lehigh University, Bethlehem, Pennsylvania, USA; 4 Division of Social and Transcultural Psychiatry, 5620 McGill University, Montreal, Quebec, Canada

**Keywords:** case discussions, community of practice, training, violent radicalization

## Abstract

Case formulation is used in clinical training to weave together theoretical perspectives and support a shared plan of action. Although a cornerstone of clinical practice, critical social theorists have highlighted the risks of depoliticizing political struggles and of reifying and fixing subjects when using psychopathology and case formulation to address situations of injustice. In the field of violent radicalization, this risk is increased by the extreme affects evoked by terror in practitioners and in societies. This article explores the challenges of training clinicians in the field of violent radicalization. It does so by analyzing a Community of Practice (CoP) that was developed to support practitioners involved in this domain of practice in Quebec, Canada. Four focus groups with CoP participants and participant observation of nine CoP meetings were conducted. Thematic and narrative analyses were used to explore the training potential of the CoP and to identify the discursive processes and group dynamics associated with this modality. Results indicate that the diversity of professional perspectives and social positionalities in the group plays a central role in helping participants become aware of their biases and in developing more complex understandings of cases and of their social embedding. Results also suggest that the collective holding of risk is key to preserve practitioners’ investment in patients involved with violent radicalization. The sensitive issue of partnership between health and social services and security agencies is also addressed. Results suggest that CoPs with strong leadership allow for experiential training to enhance clinical and critical thinking.

From Hippocratic medicine to contemporary psychology and psychiatry, case formulation has been a fundamental tool in teaching and practice (Eells, 2007). Defined as “a hypothesis about the causes, precipitants, and maintaining influences of a person's psychological, interpersonal, and behavioral problems” (Eells, 2007, p. 4), a case formulation goes beyond diagnosis and provides a more dynamic understanding of a clinical situation, weaving together complementary perspectives. In most healthcare settings, the case formulation summarizes in a coherent story what often becomes the common team understanding of a clinical presentation and evolution. In the DSM-IV's Outline for Cultural Formulation, the place of culture has been officially integrated in case formulation (Kirmayer et al., 2014) and further operationalized with DSM-5's Cultural Formulation Interview (CFI) that may become a useful tool to elicit information on the complexity of social and cultural phenomena in mental health. For clinicians, the CFI “provides a way to collect information on patients’ illness experience, social and cultural context, help-seeking, and treatment expectations relevant to psychiatric diagnosis and assessment” (Lewis-Fernández et al., 2020, p. 487). The way in which clinicians weave this information into a case formulation is, however, not a neutral or innocuous process. Influenced by context and history, it may contribute to building a culturally safe and welcoming environment for members of minority communities, or inadvertently integrate the institutionalized blind spots of the cultural majorities and reinforce stigmatization, social exclusion, or marginalization processes (Kleinman & Benson, 2006). In addition, clinical case formulations can serve to depoliticize political struggles, as psychopathology can be used to devalue the importance of environmental factors in situations of injustice (Sedgwick, 2010). This has also been the case for the notion of violent radicalization which has often been used to criminalize communities (Younis & Jadhav, 2020) and, more broadly, dissent (Toscano, 2009). Thus, training clinicians to develop case formulations when clinical situations involve violent radicalization calls for a resolutely critical approach that considers clinicians’ cultural backgrounds, social positionality, emotional responses, and ideologies in order to reflect on the usefulness but also potential harm which may be associated with clinical case formulation.

The objective of this article is to explore the challenges of developing collective case formulations in the context of clinical work with individuals involved in a process of violent radicalization. It does so by analyzing the group processes and discourses present during meetings of a Community of Practice (CoP) that was developed to train and support practitioners involved in this domain of practice in Quebec, Canada.

## Case formulation in situations of violent radicalization

There are many conflicting definitions of radicalization, violent radicalization (Kundnani, 2012; Sedgwick, 2010), and terrorism (Shanahan, 2010). In the clinical realm, defining these categories is further complexified by the fact that they have been extensively involved in specific power relations and institutionalized othering processes (Ahmed, 2020; Hörnqvist & Flyghed, 2012; Wolff, 1969). In recent years, the term radicalization has also become increasingly used as a synonym for political violence and terrorism (Bibeau, 2015). In this article, we adopt a perspective on radicalization that defines it as a dynamic process of challenging the status quo when dialogue is no longer possible (Rousseau, Miconi, et al., 2020; Schmid, 2013). As for the notion of violent radicalization, it refers to processes of radicalization in which violence is construed as a legitimate means to obtain social changes (Alcalá et al., 2017), while terrorism is defined as “actual or threatened use of violence against civilians with the aim of creating fear among other civilians as a means of achieving political goals” (Herring, 2008, p. 198).

Critical social scientists like Althusser (1995) explained how ideology produces concrete effects by creating subjects with defined roles and positions through the discursive phenomenon of interpellation. The *Oxford Dictionary of Critical Theory* defines interpellation as:

the non-coercive process whereby a subject is called upon by a particular social formation to misrecognize themselves as a subject and thereby forget that they are constituted by society rather than constitutive of society as they henceforth imagine themselves to be. Ideology recruits individuals and transforms them into subjects by persuading them to occupy a subject position it has prepared for them and see themselves in that otherwise vacant position. (Buchanan, 2018, p. 243)

The notion of interpellation is useful to understand violent political actions like terrorist acts. Some terrorist acts are targeting specific communities (e.g., Jewish, Muslim), so that all members of these communities can feel threatened by the attack through a process of interpellation. The targeted nature of the terror induced by the attack can also create a division between those who feel concerned and those who feel they are protected from this specific call.

For the question that concerns us here, i.e., the collective elaboration of clinical case formulations that involve violent radicalization, the subjective experiences and social positioning of both clinicians and those who receive care are also influenced and designed by ideologies, so that both can feel interpellated by social events.

Critical discourse analysis provides a method to gather insights on the ideological process at play during clinical group discussions aimed at formulating a case that will inform action. The task of analyzing discourses during case formulations of situations involving violent radicalization thus involves accounting for different types and layers of discourses and power dynamics present during group discussions. First, there is the professional discourse on a person that leads to the formulation of the case. This narrated story, which is both an individual narrative and a social act in the realm of discourse, is addressed and presented to a group of other professionals. Then, participants in the group can question this discourse and modify it, thus creating an interprofessional group discourse. Finally, throughout this process of discourse enunciation and transformation, power relations unfold and reproduce or challenge institutional and social discourses, such as ideology and dominant cultural representations.

Each of these discourses and levels of discourses creates different kinds of subjects, referring to a multiplicity of representations of real human beings who are not present during the discussion. These discourses are also part of specific “regimes of truth” (Foucault, 1971, 1975) that determine what can be considered as true and a real explanation of a given lived experience. Contrasting positionalities of group members, transference dynamics, and internal debates can all lead to a more complex, comprehensive understanding of a clinical situation. When these discussions are viewed through a critical lens, the resulting production is seen less as a “definite truth” and more as a co-creation of meaning around what is deemed to be “true” in a given social and relational context.

The present article presents an analysis of a novel training modality based on the development of a CoP that brings together clinicians working in five social polarization-specialized clinical teams operating across the province of Quebec, Canada. It examines the processes at play during the monthly meetings of the Social Polarization CoP, meetings which last two hours, are held through videoconferencing, and consist of either a group discussion on a common theme of interest (e.g., collaboration with security forces or risk assessment) or collectively working on the case formulation of a specific situation brought by one of the CoP members. As suggested by Wenger (1998), CoPs allow workers of a common domain of practice to exchange on topics of interest, to learn from each other, and to support one another. CoPs are also characterized by meaning negotiation and identity formation. In this article, particular attention is given to group dynamics and the elaboration of collective discourses around clinical stories, notably when risk of violence is addressed.

## Methods

### Study setting

Since 2016, an innovative intervention model has been implemented in Quebec, Canada, to provide specialized services to individuals, families, or communities who are, in one way or another, struggling with issues of violent radicalization (Ben-Cheikh et al., 2018). First implemented in Montreal, five multidisciplinary teams are currently in operation across the province (i.e., in Montreal, Laval, Quebec City, Sherbrooke, and Gatineau). These specialized teams consolidate expertise in transcultural psychiatry, community mental health and social services, and medico-legal risk assessment. Clinicians from these teams work in close collaboration with local partners (e.g., schools, youth protection, security agencies) and offer clinical follow-ups that are not mandatory and are independent from justice decisions. They work with referred individuals who are distressed or have mental health difficulties, in situations involving extremism and polarized groups, and often in a context with a high risk of violence. As there is still limited information on evidence-based practices in the field of social polarization and violent radicalization, a CoP was implemented at the same period to support clinicians from these specialized teams. The model proposed for the development of this CoP was inspired by the positive results documented on a similar group-based training modality called Transcultural Interdisciplinary and Interinstitutional Case Discussion Seminars (TIICDS). The impacts of TIICDS notably include enhanced clinical and intercultural skills, improved transference analysis capability, and prevention of professionals’ burnout (Daxhelet et al., 2018; Johnson-Lafleur et al., 2021; Rousseau, Johnson-Lafleur, et al., 2020). Discussions held during TIICDS bring new perspectives on situations and treatment, ranging from collectively reformulating the case to shifting participant personal understanding and meaning-making of the case. This is done by eliciting participants’ awareness of their implicit assumptions about the case and internalized cultural representations.

### Study design

A multi-methods qualitative study combining focus groups and participant observation was conducted between 2017 and 2019 with the overall goal of documenting the training potential of the Social Polarization CoP meetings. The inclusion criteria for participating in the study were to be part of one of the specialized social polarization clinical teams in Quebec and to attend monthly CoP meetings. Researchers obtained ethical approval from the Integrated Health and Social Services University Network for West-Central Montreal.

### Data collection

#### Participant observation

Nine CoP meetings took place over a one-year period, each of them lasting approximately 120 min. Group discussions were held in French, audio-recorded, and transcribed. All members of the CoP were proficient in French. The first two coauthors attended all CoP meetings and gathered field notes throughout the study period.

#### Focus groups

In 2018, clinicians from all locations were invited to participate in focus group discussions regarding their experience in the Social Polarization CoP. Recruitment was done on a voluntary basis and without incentive, creating a convenience sample of 17 participants from four of the five clinical teams, with an average of four participants per focus group. Researchers developed a semi-structured interview guide based on a Knowledge, Attitude, Practice (KAP) framework to gather feedback on the CoP, particularly in terms of knowledge acquisition, attitudes of participants, and perception of the CoP's usefulness as a training and supervision tool. Questions covered topics such as expectations regarding CoP meetings, overall impressions, potential impact on intervention strategies, and strengths and weaknesses of the modality. The first two authors co-facilitated the focus groups which lasted on average 70 min and were audio-recorded. As facilitators were invited to visit each team to conduct the focus groups, field notes on group dynamics were also gathered to add complementary observations to the collected material. Signed consent was obtained from focus group participants.

### Data analysis

#### CoP meetings

Critical discourse analysis (Parker, 2013) was conducted on all the narrative material to examine how it revealed group dynamics and participants’ positionality and situated knowledge. The transformation of the multiple layers of discourse along the CoP year was also examined. The first and second authors (an anthropologist and a clinical psychologist) listened to CoP meetings’ audio-recordings and read their transcripts, along with the project's field notes. Analytical and reflective notes were taken about each session and about the CoP year as a whole. These notes were first compiled individually and then discussed to reach a consensus. The results of the analysis were then reviewed by coauthors who are familiar with the field to ensure they reflected the content of CoP sessions.

#### Focus groups

Data were analyzed using thematic content analysis (Boyatzis, 1998). First, analytical and reflective notes were taken while listening to the audio-recordings and were discussed between the first two authors. Then, a codebook was created by going back and forth between a priori (interview questions) and emerging categories, the audio material, and the notes. Thematic saturation was reached after all focus groups had been analyzed and no more codes emerged from the data. The codebook was applied to all the material. Final themes included global satisfaction, expectations, challenges, and impacts of training. Finally, all coauthors were invited to critically review and to refine the results of the analytical process.

## Results

### Participants’ perception of CoP meetings

Focus group findings indicate that participants reported a high sense of general satisfaction, stating that CoP meetings were an efficient training practice. Participants found them to be “nourishing,” “interesting,” “helping in motivation,” and a “great source of support.” Participants also appreciated the sharing of expertise across different geographical regions and between participants. Besides receiving theoretical and clinical training on social polarization and violent radicalization, participants’ expectations of CoP meetings also included having access to support from the Montreal team and being taught about the lessons learned from previous clinical cases on radicalization. In terms of challenges, participants reported that dealing with technology was difficult. Since CoP meetings were held through videoconferencing links among five sites, participants only had access to small screen images of the other teams which greatly minimized non-verbal communication. Participants also shared experiences of institutional difficulties in legitimizing the time spent in CoP meetings to their administrative directions. The partnership with security forces was also underlined as challenging because of the delicate balance between establishing working alliances and maintaining strict confidentiality. Beyond the strict limits provided by the Quebec law that do not allow confidentiality breaches except in situation of immediate risk for life, the CoP meetings seemed to help define professional boundaries in a context of high risk and provide resources on how to establish meaningful partnerships with security agencies, while enforcing robust firewalls. In brief, the perceived impacts of the CoP meetings were of three kinds. First, they provided a complexified understanding of certain situations and shed light on transcultural issues and on risk assessment. Second, they enriched specific intervention plans and increased the participants’ trust and confidence in their work. Finally, they reinforced the participants’ position on the importance of preserving a separate clinical space in front of legal authorities.

### Group dynamics and discursive processes during CoP meetings

We will now present results from the analysis of the content of CoP meetings by focusing on the cases of three specific meetings that have been selected to illustrate the chronological evolution of the group dynamics and discursive content: the first session, one session held at mid-year, and the final session (see Table 1).

Among the discussions held during these CoP meetings, the following four overarching themes were identified: “constructing group identity,” “dialogical deconstruction,” “subjectivity and interpellation,” and “dealing with danger and risk.” These themes will now be presented chronologically, within the context of the CoP meeting in which they emerged.

### Constructing group identity

The first moments of the very first CoP meeting were used to introduce ground rules and establish a trust-based contract between the members of the group. The facilitator started by welcoming the participants and then proceeded to set out rules to be followed for successful collective work, notably in terms of mutual respect. It is important to note that this first session was led by the Montreal team and was facilitated by a senior psychiatrist with implicit authority in the group. The facilitator's symbolic function as the one who spoke first and set out the rules appeared as crucial for this first meeting. It seemed to contribute to the development of a sense of holding for the group and somewhat of a form of protection from the outside world. For example, when the facilitator clarified the rules about “confidentiality,” it seemed to bring a safer atmosphere, as documented in our field notes and illustrated in the following extract:

To start, we need to define the framework […] and some fundamental principles, and the first and most important one is confidentiality. This confidentiality must be extremely strict. […] We trust you […] but be very careful about the confidentiality. […] It's our only absolute requirement.

The construction of the group's identity was possible to examine through both focus group content and participant observation. Most of the participants were new to this type of setting and were discovering the possibilities offered by such a group. Many participants mentioned that they did not know what to expect of these meetings but then gradually started to feel a sense of belonging to a community, that of professionals working in the field of social polarization and violent radicalization. In terms of the development of group identities, the local teams’ identities followed various temporal trajectories and were influenced by specific regional contexts.

I have the impression that you are likely to go through the same phases in the different teams as we did [in Montreal]. Beginning with a dormant phase where you’re basically asking yourself: is it really worth having a team and is there really a demand? – Yes. We’re in the middle of that. We can’t wait for it to start.

These processes of identity development have been observed during the CoP year during which both a global CoP identity as well as local teams’ identities have been constituted, creating a group identity as well as a diversity of voices, concerns, and power dynamics.

### Dialogical deconstruction

Based on the recognition of the co-existence of multiple and contradictory voices within the group which can creatively enter into a dialogue, we use here the notion of dialogical deconstruction to refer to the process of questioning one's individual perceptions and generalization, and of facilitating the integration of alternatives ideas. This was observable during the first CoP meeting and is illustrated in the following extract during which a psychologist presenting a case seeks to reassure the other participants regarding the risk of violence presented by a young man he sees in the clinic:

Psychologist: “He is someone I have invested a lot in and very quickly. So, I have a lot of empathy for this young man. I think things are going well.”

Facilitator: “What is striking me the most, as an outsider of this therapeutic space, is that [Name of the psychologist] is attached to this youth. Whereas he can rather appear as a terrorizing youth. He terrorizes everyone. This tells us something about the enormous emotional needs of this youth but also about his attachment capacity. He is extremely endearing […] but [Name of the psychologist] is going to minimize his threatening aspect.”

Psychologist: “I am not worried because of what I see … He does not have the profile of a psychopath this young man.”

Participant A: “You said he has a tendency of splitting between the stakeholders. The notion of risk can also be perceived differently from one stakeholder to another.”

Participant B: “How far can we go in prevention? I think there are a lot of things that have been done to date, but I also think we have to be, in my opinion, and it's my personal opinion, a little more active in terms of medication since the ratio between benefits and risks is, I would say, pretty good in this situation.”

Facilitator: “This is indeed a case we would like to discuss with an external consultant to better assess the risk. Not because we are not terrified by this youth, but rather because we are preoccupied by the fact that we are *not* terrified while there are other people in the network that really are.”

During this discussion, different discourses and regimes of truth were deployed, illustrating how risk is assessed differently by different protagonists and can be based on power dynamics, as well as relational and transference elements.

### Subjectivity and interpellation

The second case took place in the middle of the first CoP year and included a case presentation connected to the terrorist event that occurred at a Quebec City Mosque in 2017 where six worshippers were killed and 19 injured. This session was an opportunity to observe the group in the face of a collective trauma and see how participants’ identities can lead them to take different positions and therefore to be “called out” or “interpellated” differently, being more or less challenged by an ideology and by emotional and personal elements.

Our observations suggested that not all participants were challenged in the same way by this case, or “interpellated,” as illustrated for instance by the fact that many participants had some difficulty “naming” the terrorist. One participant stated: “Perhaps just to clarify that … services were also provided to the family of the person who … the accused in fact, the person who killed.” At the time of this meeting, the perpetrator of the attack had not been legally recognized as a “terrorist,” and the group discussion highlighted that some people did not seem comfortable calling the young man in question a “murderer.” Instead, they used expressions such as the “accused person,” or they called him by his name, “Alexandre.”

Some meeting participants talked about the Muslim community with referents such as “this community” or “among themselves” which created a certain distance through a process of othering. There are many possible explanations for this phenomenon, one of which is that the othering process protected these participants against the traumatic event by putting distance between them and the victimized group. This, to a certain extent, also had the effect of locating the conflict in the outside world and far away. Another hypothesis is that in this case, the othering process could also represent a way to refuse this “interpellation,” and so to refuse to be called out by the terrorist attack as “subject-victim” of the white supremacist ideology.

Some discourses heard during the meeting also raised the question as to whom can be identified as legitimate victims in certain social discourses. For instance, one victim of the attack was considered as not performing his expected role of the “absolute victim” by using the compensation money to repair his house:

[O]ne person who was shot … I think this person decided to repair his house. And this raised a lot of questions for the treatment team. […] We often heard, and we are still hearing, some discourses such as “How is it possible to give them [the victimized families] this much …” – and I put this between quotation marks – “to these families, whereas I see a lot of Quebec families who are also in need.”

Another discourse present in the meeting appeared in some way as mirroring or repeating the terrorists’ discourse. The social discourse that portrays Muslims as the aggressors from whom the larger community must be protected is crucial to understand the difficulty experienced by participants when referring to the perpetrator as a “terrorist.” This discourse, as noted by the facilitator, carries an implicit double standard: “It is a double standard. […] It is a terrorist attack if the perpetrator is a Muslim person so it should be too if the perpetrator is Quebecois.” Other CoP participants further challenged this implicit social discourse and created a sense of solidarity with the victims through a common interpellation by far-right terrorism. In other words, the discussion started with a clear distinction between the Muslim Community and the Quebecers, but then, some Muslim and other non-white Quebecers showed the complexity of the Quebecer identity with their own positionality. For instance, two participants initiated their narrative by positioning their identities, stating, “You know, we are two Muslims persons,” then invited the group to introduce more complexity in their reflection by saying:

There are families in Quebec […], they migrated. But they are here since a long time ago. They are part of the society. […] What we are trying to tell you is to try not to refer to the Muslim community in terms of “us” and “them.” It's an inclusive “us.”

Another participant joined in, adding:

This is true. We say: it's difficult for “them,” but in fact, it's difficult for all of us what happened. […] I feel the terrorist attack of Quebec City is terrible. It is so for me personally, as a clinician and as a human being.

After the discussion and self-reflections regarding biases toward different communities, the group began to address some of the blind spots of Quebec institutions. The lack of understanding of the collective trauma and its active denial and avoidance, along with the complex responsibilities associated with the Quebec City attack, were emphasized. During the meeting, it was possible to see how the presence of different discourses contributed to the co-construction of a more complex appraisal of the situation which integrated individual experiences and different levels of discourse. This complexification included paying attention to the host society and its institutions, and addressing the historical and societal basis of structural discrimination. The diversity of points of view and the diversity of people composing the group contributed to a sense of collective empathy, as each participant could be seen as the Other of another one.

This CoP meeting was also an important turning point in the group dynamic: it seemed to challenge the illusion of a totally safe group, an impression created at the beginning of the CoP meeting series. Participants started to claim their individuality or regional specificity. Observing this dynamic also revealed internal tensions in the group. It was also a turning point for the Montreal team who needed to acknowledge the demands and identities of other regional teams and legitimize the diversity of representations. Because this meeting was about a shared trauma that affects individuals as well as larger communities, the observed tendency of fragmentation in the group could be understood as a re-enactment, on the collective scene, of the societal division provoked by the terrorist attack. In this session, the wider social discourses were strongly influencing the microenvironment of the CoP and weakening, to a certain extent, the “safe-enough space.” Some participants were reenacting the aggressors, while others were identifying with and embodying the targeted communities.

### Dealing with danger and risk

The third case presented in this article was the final CoP meeting of the year. This closing session can be read as a variation on the theme of the opening session. The aim of the opening session was to establish a “safe-enough space” in which CoP members could work without feeling too threatened. Interestingly, at the request of participants, the theme discussed during the last CoP meeting was that of security and, more specifically, how to deal with both individuals at risk of violence and security forces. Risk assessment is an important part of the work when dealing with violent radicalization, although the CoP do not explicitly aim to teach risk assessment, as the Montreal team provides distinct workshops on this topic.

The meeting started with a reminder by the facilitator about the health professionals’ codes of conduct and ethics regarding confidentiality: “What do we say to security forces? We say nothing.” The group freely talked about their worries and about how they find it difficult to deal with risk. Some participants asked for the group to help them to hold the risk:

Someone said “[we know] we have to take the risk on the clinical level” […], but we found out it is a road … a bit … a rocky road. I hope that … well […] we can count on your support and advice too.

The facilitator also used the strength of the group to reassure the participants and propose a collective way to assume the risk:

I think that the best way to feel at ease with the risk is to share it. […] By doing this, we carry the risk together. Instead of turning to the rationale of “I only followed orders” used by security forces, we can say, “We collectively made this decision.”

Different experiences presented throughout the CoP year can show the complexity of building partnerships between different sectors, notably between the health and social services and security sectors. The experiences reported during the CoP meetings have hinted that the implication of security forces is a delicate matter that can sometimes be done against the clinical mandate of health and social services. The alternative offered by the Social Polarization CoP is to build a collective sense of responsibility, relying on the group's strength to make decisions when working with high-risk situations.

## Discussion

### Creating a community of practice to foster experiential learning

Results from this study indicate that the Social Polarization CoP offers a critical experiential learning tool to improve clinical and critical thinking when dealing with situations involving violent radicalization. Teaching clinical thinking (Fuks et al., 2009) is challenging because it encompasses all the relational, implicit, and interactional elements that structure clinical practice. Learning from experience and affects entails going beyond a limited account of descriptive behaviors and attempting to understand the lived experience of the subject. Group dynamics, along with group supervision, can create an affective community of learning that offers the possibility of experiencing and establishing links between affect and cognition (Sandor, 2014). Such affective community, framed by collective implicit and explicit rules, can create a safe enough space (Johnson-Lafleur et al., 2019) for critical teaching (hooks, 2010). Some conditions of the CoP meetings facilitated a critical reflexive learning environment, notably their interdisciplinary and interinstitutional format. This characteristic of the CoP challenges some hierarchies and in doing so values subaltern knowledge through openness in dialogue. The CoP also aims at addressing, and at times challenging, the contextual, environmental, and political factors involved in the presenting situation and therapeutic relationship. Discussions can reveal how racism, colonialism, and other social determinants shape our understanding of situations due to our differentiated positionalities, predicaments, and privileges. The lived experience of the CoP thus creates an opportunity for experiential critical learning by integrating multiple perspectives.

### Unlearning certainties and enabling ethical complexity

Engaging in dialogue offers the possibility of “unlearning” certainties and to be critical and creative. Such a process of dialogical deconstruction in a reflexive community is the main goal of the Social Polarization CoP. However, creating a meaningful dialogue remains a challenge during CoP sessions, as the reproduction of ideological discourses rooted in dominant social norms is often found in the implicit shared values of groups, including the Social Polarization CoP. Complexified discourses can nonetheless be enabled and created through empathy and recognition. If we come back to the words of the psychologist from the first case—“I am not worried because I see …”—we can read these words as illustrating the recognition of the other through the ethical encounter with the “face” of the other (Levinas, 1984). It is an ethical move that goes from looking at something, an object, to sharing a responsibility by acknowledging the humanity (the face) of someone else. The collective sense of reality is born from such dialogical conflicts between, on the one hand, face-to-face recognition, and, on the other hand, othering and excluding discursive processes. For example, chemical restraint appears sometimes to be a way for professionals to control their anxiety in front of potential risk of violence, as exemplified when a participant suggested, “to be more active in terms of medication.” However, discourses are partial accounts of reality. In this case, the discourse highlighted the risk of an individual to harm but did not account for other affective, relational, and political elements.

### Subject-making and interpellation: Situated perspectives on violence

The field of violent radicalization is highly political, and clinical work in this domain requires a strong ethical stance and clear professional boundaries. Our research results have showed how the relationship between professionals and the people they provide care to impacts the development of the professionals’ formulation of the case. The positionality of professionals also has a strong influence on the perception of the case.

Phenomenology (Husserl, 1966) argues that our perceptions are judgments influenced by social constructions. What we think of as a neutral perception of a clinical case is in fact based upon perceptions filtered through social and scientific categories. Personal history and social position also influence perceptions. For example, the perception of violence often varies depending on our social standing and class (Dorlin, 2017). It also varies depending on whether we are suffering from systemic violence or benefiting from this system. Having a diverse group in terms of social positionalities is an effective way to explore different perspectives and discuss unquestioned social norms.

The case on the Quebec City Mosque attack is exemplary in showing that diversity of knowledge is linked to diversity of perception and positionality. Participants identifying as members of the majority of Quebec society and participants from minority communities were involved in discussions that are difficult to have in the social space. However, these types of discussions contribute to counter social polarization by allowing a critical and open dialogue. The creation of a safe enough space with the illusion of being protected from the outside world, including from institutional violence, makes a critical dialogue possible to develop a complexified and enhanced case formulation. Knowledge is produced by and located in different communities, and members of these different communities can bring to the group key elements to the development of the case, such as the feeling of being the specific target of a given discourse. The attack of the Quebec City Mosque raises important questions for Quebec society: can the perpetrator represent a figure of evil as the jihadists typically do in dominant social discourses? As a white Quebecois he is part of the majority, which means that in this case, the othering process is less active for professionals from the majority. He is also a young White male, in some way symbolizing privileges that “should not” be attacked, under the wider social regimes of truth.

Overall, different positionalities and forms of subjectivities were called out (interpellation) during the CoP discussions throughout the year. For example, gender diversity in the group was important to better understand processes of case formulation on situations involving gender issues (e.g., masculinist groups), and religious and ethnocultural diversity was important to better understand situations related to the threat of the far right. Situated knowledge highlights the importance of a diverse group to develop a broader inclusive case formulation. Processes of individual and collective transference make the CoP an effective strategy for experiential learning, providing the opportunity to work with and work on positionalities, and to account for situated knowledge. CoPs as a training modality provide participants a sense of diversity: diversity in ways of seeing the world, diversity of emotions related to social events, and diversity as a way to cope with collective traumatic events.

### Collective holding of risk, affect, and responsibility

Tools and scales for risk assessment provide important guidelines and expertise to professionals dealing with violence but fail to teach clinical judgment. Our results suggest that the Social Polarization CoP is an effective training modality to teach how to deal with violence threats by combining the use of tools, clinical judgment, and group dynamics. Its format provides a lived experience of collectively sharing a sense of both reassurance and ethical responsibility through collectively reclaiming power as a group. Violence threats are also two-sided and bidirectional. They can consist in potentially receiving or potentially causing harm. The Social Polarization CoP tries to avoid both of these extreme stances by collectively assessing risk and providing boundaries. It also tries to avoid harm by refusing to profile cultural minorities and to perform risk assessment based on fear and ideologies of exclusion. Collective case formulation also helps to regulate affects and to create a more complex view of clinical situations, as illuminated by the different areas of practice and expertise represented by the entire CoP membership.

### Limitations and implications

This research has several limitations given the innovative and sensitive nature of the practice studied, as well as the positionality of the first two coauthors who conducted the data collection activities. Qualitative methods have the advantage of taking subjective biases into account but can only provide a partial perspective on the phenomenon being studied. The position of the first two authors was presented to research participants as that of independent researchers. Nevertheless, most of the participants considered them as members of the Montreal clinical team, as induced by their presence in the videoconference room in the Montreal setting. This likely influenced what was discussed and named or not during the focus groups.

Another important limitation of this study is that it was conducted during the early stages of the Social Polarization CoP development. As such, further research is needed to assess if this training modality has a positive impact on clinicians in the long run and on their interventions’ outcomes. In addition, the feasibility of reproducing such a training modality is limited considering the many conditions that are needed for it to be efficient. First, it requires a facilitator with both a clinical and research background as participants are looking for both clinical supervision and teaching on best practices informed by research. Second, it requires participants who are willing to create a safe enough environment and allow everyone to express themself freely and in a confident manner. Finally, the CoP requires a diversity of social positionalities (i.e., religion, gender, social class, etc.) and professional ideologies (i.e., professions, organizations, therapeutic models, etc.) among participants to enrich the discussions. These conditions are not easy to implement and are important limitations in creating other groups with the same dynamics.

Despite these limitations, the results of this study can contribute to further reflection on the possible drifts of clinical work in the field of social polarization and violent radicalization, and on the need to support and train professionals who are on the front lines of these issues.

Overall, our results indicate that the process at play during the Social Polarization CoP meetings contributes to both deconstruct and formulate clinical cases. Shedding light on how knowledge is constructed and repressed, avoiding essentialism and determinism, and enabling complexity-building through critical dialogue are at the heart of the Social Polarization CoP initiative. Every failure collectively experienced, and every step taken to repair relationships and the world, is also an important experience for understanding and accepting that ideal communities do not exist. The potential of the Social Polarization CoP lies in and relies on a group that is strong enough to collectively be part of a clinical adventure, while remaining aware of its limitations and potential failures. CoPs are the art of understanding together and supporting each other in the face of difficult realities, thus contribute to preventing disengagement of clinicians and minimizing the retaliation toward those who receive professional clinical attention.

**Table 1.** Three CoP meeting sessions selected for analysis.

**First case** This CoP meeting was the first to be held after the creation of the Quebec specialized clinical teams on social polarization and violent radicalization. Participating in the CoP was thus a novel experience for most members of the group. A case discussion was presented by a psychologist from the Montreal team. It led to a rich discussion on clinical work in this field and raised questions on issues of transference, attachment, and risk assessment. The case was about a young man with an identity conflict between his belonging to a cultural minority and his fascination with extremist groups. He watches and expresses hate content online.
**Second case** During this meeting, the Quebec City team presented how they were solicited following the terrorist attack that occurred against the Muslim community in Quebec City. The team described the social environment and the different struggles they encountered in the aftermath of this collective trauma. This CoP meeting occurred during the healing process related to these traumatic events, and it led to a variety of discourses and reactions. The meeting was particularly interesting as it pertained to a more collective situation rather than to a specific individual intervention.
**Third case** This session was the last CoP meeting of the year, and the teams chose to discuss topics they found challenging in the field. Part of the discussion focused on establishing limits when working with security forces within a psychosocial mandate, and risk management and assessment.
